# MRI-based quantification of adipose tissue distribution in healthy adult cats during body weight gain

**DOI:** 10.3389/fvets.2023.1150085

**Published:** 2023-05-05

**Authors:** Francesca Del Chicca, Henning Richter, Hans-Peter Müller, Jan Kassubek

**Affiliations:** ^1^Clinic for Diagnostic Imaging, Department of Diagnostics and Clinical Services, Vetsuisse Faculty Zurich, Zurich, Switzerland; ^2^Department of Neurology, University of Ulm, Ulm, Germany

**Keywords:** MRI, feline, overweight, hepatic fat fraction, visceral fat, subcutaneous fat

## Abstract

The incidence of obesity in pet population increased over the last decades. Cats have been suggested as model for human obesity because of similar co-morbidities as diabetes and dyslipidaemia. Aim of this study were to quantify the distribution of visceral and subcutaneous adipose tissue (VAT, SAT respectively) in healthy adult cats during feeding-induced body weight (BW) gain by MRI, and to correlate it to the increased hepatic fat fraction (HFF). Cats received a commercial dry food *ad libitum* for 40 weeks and were longitudinally scanned three times. VAT and SAT were determined from Dixon MRI data by a dedicated software solution (*ATLAS*, established in human and rodents). HFF was quantified from a commercially available sequence. At both individual and group level, normalized adipose tissue volumes significantly increased longitudinally, with median VAT/SAT ratio always < 1. With increased BW, more than proportional increased total adipose tissue was observed together with more than proportional increased HFF. HFF is disproportionately high in overweight cats compared to SAT and VAT accumulation in the 40 weeks observation period. Quantitative unbiased MRI examination of different body fat components is useful in longitudinal monitoring of obesity in cats.

## 1. Introduction

Overweight and obesity are defined as deposition of excessive adipose tissue in the body (1, 2). Companion animals are defined overweight when they are between 10 and 19% over the ideal body weight and obese when more than 20% (3). The prevalence of overweight and obesity in cat population is reported up to 63% (4) and, similarly to humans, the incidence of obesity in pet population has increased over the last decades (5). Several diseases are associated with obesity in cats, like diabetes mellitus and reduced insulin sensitivity (6), hepatic lipidosis (5), and a variety of orthopedic, cardiac, respiratory and urogenital disorders (1).

In humans, visceral adipose tissue (VAT) including pericardial adipose tissue has been reported to be linked to vascular inflammation and insulin resistance, while the role of the subcutaneous adipose tissue (SAT) is still ill-defined (7). Distribution of VAT and SAT has been quantified in humans based on whole-body T1-weighted MRI in different studies (8, 9). Other studies use T1-weighted (10) or two-point Dixon images (11) of the abdomen only. Moreover, different MRI-based methods are used to quantify hepatic fat fraction (HFF), which, together with the VAT, is a very strong determinant in insulin sensitivity (12).

Cats have been suggested as model for human obesity (13) because of the similar co-morbidities like diabetes and dyslipidaemia. In cats, however, only few studies investigated the adipose tissue distribution. Computed tomography images of the abdomen have been used for the assessment of VAT (14) and VAT/SAT ratio (15). No difference has been found between amount of VAT and SAT distribution in lean and obese cats based on MRI of the abdomen only (6). In the available literature, the fat tissue distribution has been analyzed in each cat only on a single time point. The increase of HFF during body weight (BW) gain has been described based on a recently commercially released multiple echo gradient recalled echo (GRE) sequence (Philips mDIXON-Quant) (16). Postprocessing of the multiple echo GRE sequence for HFF quantification has been reported (16). However, no study investigates, so far, the whole-body (intended as thorax and abdomen) distribution of VAT and SAT in cats and the distribution change during BW gain over time, neither the correlation of the fat deposition with the HFF, measured non-invasively from MRI images.

The aims of the present study were the following: first, to investigate the feasibility of quantifying VAT and SAT from whole-body (intended as thorax and abdomen) MRI images in cats, using and established method in men (17) and rodents (18); second, to investigate the distribution of VAT and SAT in healthy adult cats after a period of BW gain and after a period of BW stabilization by MRI; third, to correlate the increase of VAT and SAT to the increase of HFF (16) during the period of observation.

## 2. Materials and methods

The prospective, experimental study was approved by the Cantonal Veterinary Office, ethics committee, license number ZH118/16, was conducted in accordance with Animal Welfare Act of Switzerland as part of a larger concurrent study (19), and is reported in accordance with ARRIVE guidelines. Cats were acquired as kittens, years before, as research animals from a breeding station for research animals (Liberty Research Inc., Waverly New York 14892, United States). Cats underwent MRI examination at 3 time points: baseline (at the start of the study before dietary intervention) and twice after the start of dietary intervention (follow-up 1 and follow-up 2, each 20 weeks apart).

### 2.1. Animals

Twelve adult, male, neutered shorthair cats bred for research purposes were enrolled in this study. The median age of the cats at the beginning of the study was 77 months (range 75–78 months). All cats underwent a clinical examination. Based on physical examination, hematology and biochemistry, all cats were deemed to be in good health, except two cats (cat number 8 and number 10) with mild elevation of the renal values (International Renal Interest Society, IRIS state 2) that were classified as American Society of Anaesthesiologist II. Cat number 8 has creatinine value of 176 μmol/L, and cat number 10 of 200 μmol/L (reference values 98–163 μmol/L). Cat number 10 was excluded from the study before the first follow-up MRI examination, and cat number 8 before the second follow-up examination due to causes that are not related to the study. They both were excluded from the statistical analysis.

The BW of the cats was recorded before every MRI examination. During the first week of dietary intervention, the daily ration was stepwise increased. In particular, during the first 2 days each cat was fed the resting energy requirement (19) increased by 50%. The daily resting energy requirement was doubled on day 3 and 4. On day 5 and 6 the daily ration was increased to 2.5-fold of the resting energy requirements. From day 7, dry food (Hill’s™ Science Diet™ Adult Optimal Care, Hill’s Pet Nutrition) was provided *ad libitum* for a period of 40 weeks (19). Follow-up 1 was performed 20 weeks after the beginning of the study as documentation of BW gain and before a second period of equal length of BW gain stabilization.

The total study length was 40 weeks. Water was available *ad libitum* during the entire study and daily check of food uptake was performed. After the completion of the research study, the cats were re-homed by private owners and did not participate in any further research.

### 2.2. Anesthesia

The cats were fasted for 12 h before anesthesia. Premedication consisted of ketamine (10 mg/kg), midazolam (0.1 mg/kg) and butorphanol (0.3 mg/kg) intramuscularly. After premedication a catheter was aseptically placed in the left or right cephalic vein for administration of contrast medium, intravenous medication as well as Lactated Ringer’s solution (3 mL/kg/h). Oxygen was administered via a facemask for 30 min prior to anesthesia induction. Anesthesia was induced with alfaxalone (0.5–2.0 mg/kg) intravenously. After induction the cats were intubated with a cuffed endotracheal tube and mechanically ventilated with positive-pressure in a pressure-controlled mode (5–11 cm H2O). The respiratory rate was adjusted to achieve an end-tidal CO2 of 35–42 mmHg (4.7–5.6 kPa). The anesthesia was maintained using isoflurane together with a 1:1 ratio of oxygen and air. Anesthesia was monitored and recorded with a multiparameter monitor that included spirometry, capnography and an MRI-compatible wireless respiratory sensor, as well as vectorcardiography and pulse oximetry. Glycopyrrolate (10 mcg/kg, intravenously) was administered, if the pulse rate fell below 100 bpm for longer than 10 min. If necessary, this procedure was repeated once.

### 2.3. MRI protocol

All cats were placed in dorsal recumbency in a 3 T MRI (Philips Ingenia 3.0 T scanner, Philips AG, Zurich, Switzerland). MRI examination included morphological images to exclude abnormalities. Performed sequence were: T2-weighted of the abdomen (turbospin echo; TR/TE 2,000/80 ms; flip angle, 90°; FOV adapted to animal; in-plane voxel size 1.2 × 1.4 mm^2^; slice thickens 3.0 mm; no gap) and total body T1-weighted pre-contrast sequence (mDixon, gradient echo; TR/TE1/TE2, 3.7/1.2/2.4 ms; flip angle, 10°; FOV, adapted to animal; in-plane voxel size 1.5 × 1.5 mm^2^; slice thickness 3.0 mm; slice gap 1.5 mm), from the thoracic inlet to the pelvis.

For the fat quantification, a proton density fat fraction (PDFF), multi-echo acquisition, multi-peak mDixon sequence with T2* correction was performed of the cranial abdomen on transverse plane (mDixon-Quant, Philips AG Healthcare, Zurich, Switzerland). The following sequence parameters were used: breath hold, expiration; TR/TE1/TE 7.5/1.2/1.0 ms; flip angle, 3°; FOV, adapted to animal; slice thickness 4.0 mm; slice gap 2.0 mm; acquired voxel size 1.5 × 1.5 × 4.0 mm^3^; six echoes. Breath hold technique was used for maximum 21.3 s. Therefore, controlled mechanical ventilation was discontinued to force brief expiratory apnoea and was continued immediately after the sequence.

To exclude morphological abnormalities, T1-weighted post contrast sequence was performed after hand injection of contrast medium (Gadodiamid, GE Healthcare AG, Glattbrugg, Switzerland; 0.3 mL/kg, intravenous) followed by a 10 mL saline (0.9%NaCl) solution as the pre contrast sequence (mDixon, gradient echo; TR/TE1/TE2 3.7/1.2/2.4 ms; flip angle, 10°; FOV adapted to animal; in-place voxel size, 1.5 × 1.5 mm^2^; slice thickness 3.0 mm; slice gap 1.5 mm). T1 sequences, pre and post contrast, were acquired in coronal orientation.

### 2.4. MRI data processing and data analysis

**Figure 1:**
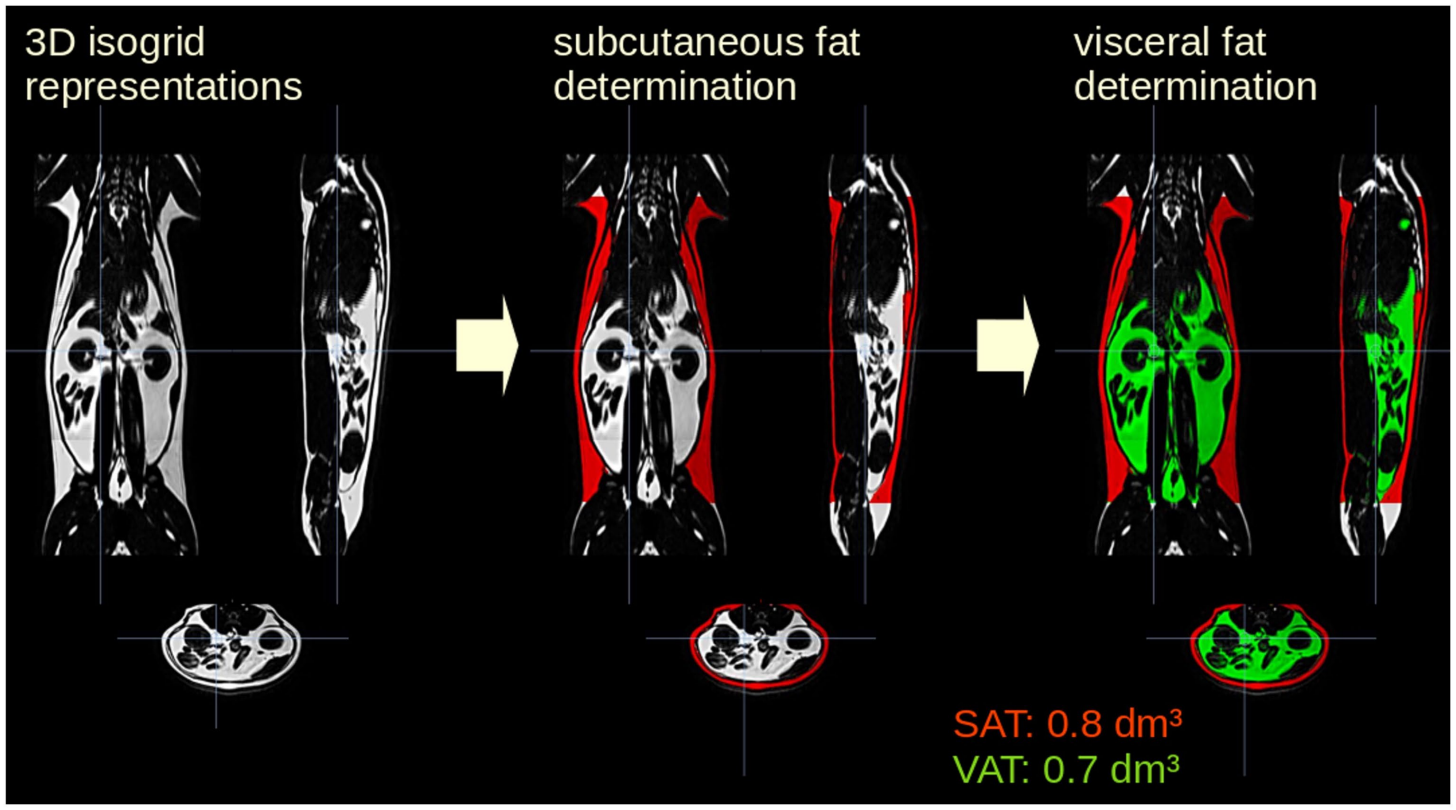
Analysis postprocessing steps for one representative cat included in the study. The data were supersampled to isotropic voxels. Subcutaneous adipose tissue (SAT, red) determination was performed using the *ARTIS* algorithm (Adapted Rendering for Tissue Intensity Segmentation); visceral adipose tissue (VAT, green) was identified by selecting all connected voxels with respect to their intensity within the from ARTIS predefined range.

**Figure 2:**
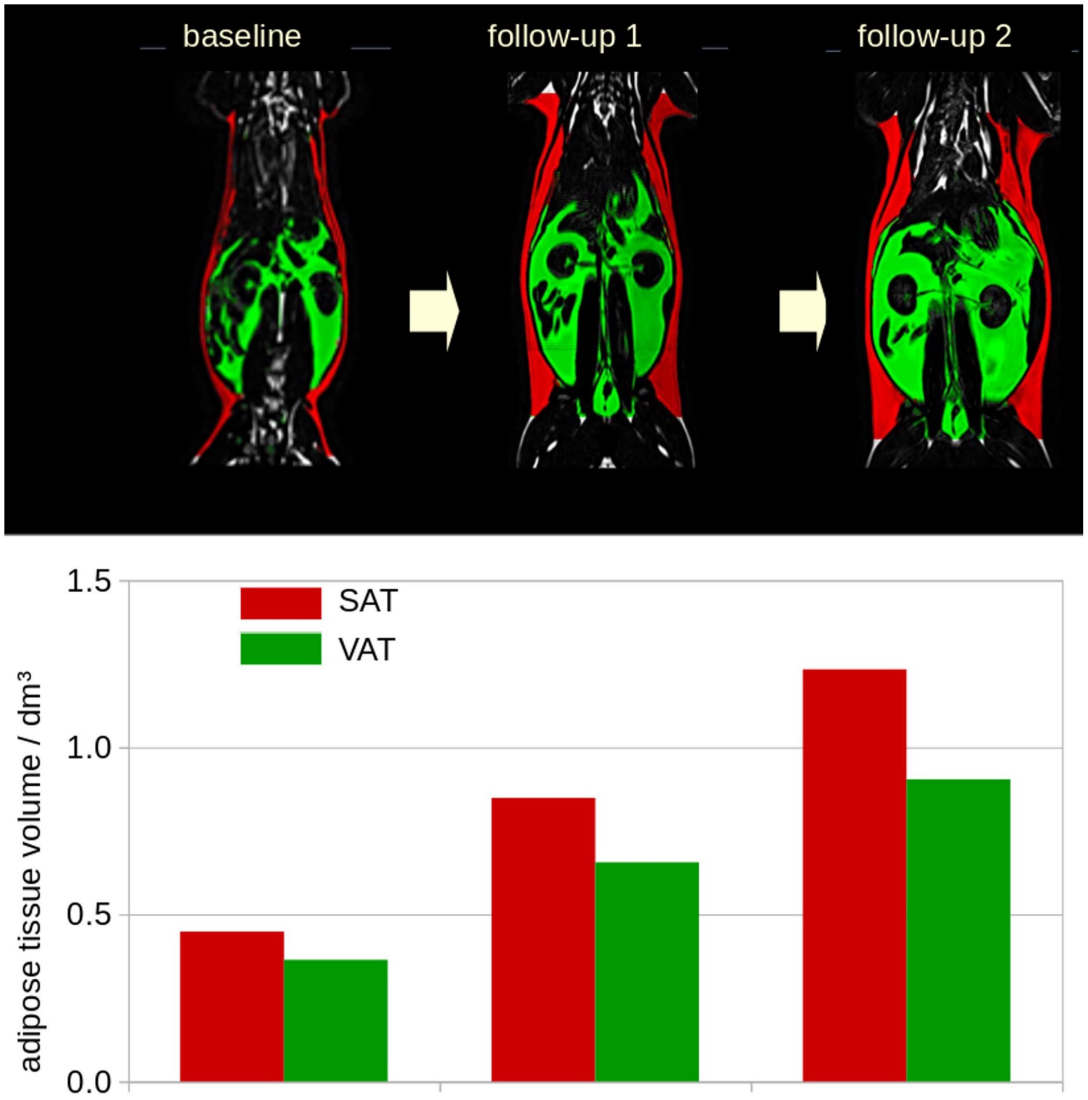
Example of the longitudinal development for one representative cat included in the study. Longitudinal scans were analyzed sequentially with a visual determination of the analyzed body area. Subcutaneous adipose tissue (SAT, red), visceral adipose tissue (VAT, green). dm^3^: 0.001m^3^.

Data pre- and post-processing was performed by the in-house developed software package *ATLAS* (Automatic Tissue Labelling Analysis Software) (17) (Figure 1): prior to subsequent analysis, the data were supersampled to isotropic voxels. In order to homogenize intensity in the data sets, unsharp mask filtering was applied. SAT determination was performed using the *ARTIS* (Adapted Rendering for Tissue Intensity Segmentation) which has already proven to reveal high stability in the results. As a special modification to improve adipose tissue determination in thoracic regions, the depth of subcutaneous fat detection was increased. Abdominal VAT was identified by selecting all connected voxels with respect to their intensity within the range predefined by the ARTIS algorithm. Longitudinal scans were analyzed sequentially with a visual determination of the analyzed body area. In addition, results of different scans of one cat were visually controlled and manually corrected in a parallel analysis setting (Figure 2). For the quantitative evaluation of the HFF, 4 regions of interest of similar size were manually drawn on the liver parenchyma using an adjustable round cursor on the automatically generated fat fraction images. The regions of interest were drawn in the right cranial, right caudal, middle, and left aspects of the liver parenchyma. In all image processing, care was taken to avoid major blood vessels, the gallbladder, and obvious image artifacts when placing the region of interest (16).

### 2.5. Statistical analysis

Because of the small study population and the non-normally distributed data, numerical data were reported as the median ± interquartile range. Differences at the group level were calculated by Mann–Whitney *U*-test and associations were calculated by Spearman rank correlation for non-parametric data (in case of small sample size) by Pearson correlation.

## 3. Results

Twelve cats were examined at baseline, 11 at follow-up 1, and 10 at follow-up 2. For the following analysis, only the 10 cats with all three scans were included. Reliability of the analysis was tested by intra-operator variability of 8 data sets, receiving a coefficient of variation < 5%.

### 3.1. Body weight examination by weighting and HFF

Median BW (of the 10 cats with all three scans) was [median (interquartile range)] 4.6 (0.3)kg at the baseline, 6.4 (1.1)kg at follow-up 1, and 6.5(1.2)kg at follow-up 2. There was median increase of BW at the group level of 39% between the baseline and follow-up 1, and median increase of BW at the group level of 2% between follow-up 1 and follow-up 2. Overall, the median BW showed an increase of 41% between baseline and follow-up 2. The median measured HFF was 3.0(0.6)% at baseline, 3.2(1.0)% at follow-up 1, and 4.7(2.1) at follow-up 2. At the group level, the median HFF increased by 7% between the baseline and follow-up 1, and by 32% between follow-up 1 and follow-up 2, respectively. Overall, the median HFF increased by 41% between baseline and follow-up 2 at the group level.

### 3.2. Adipose tissue determination by MRI

**Figure 3:**
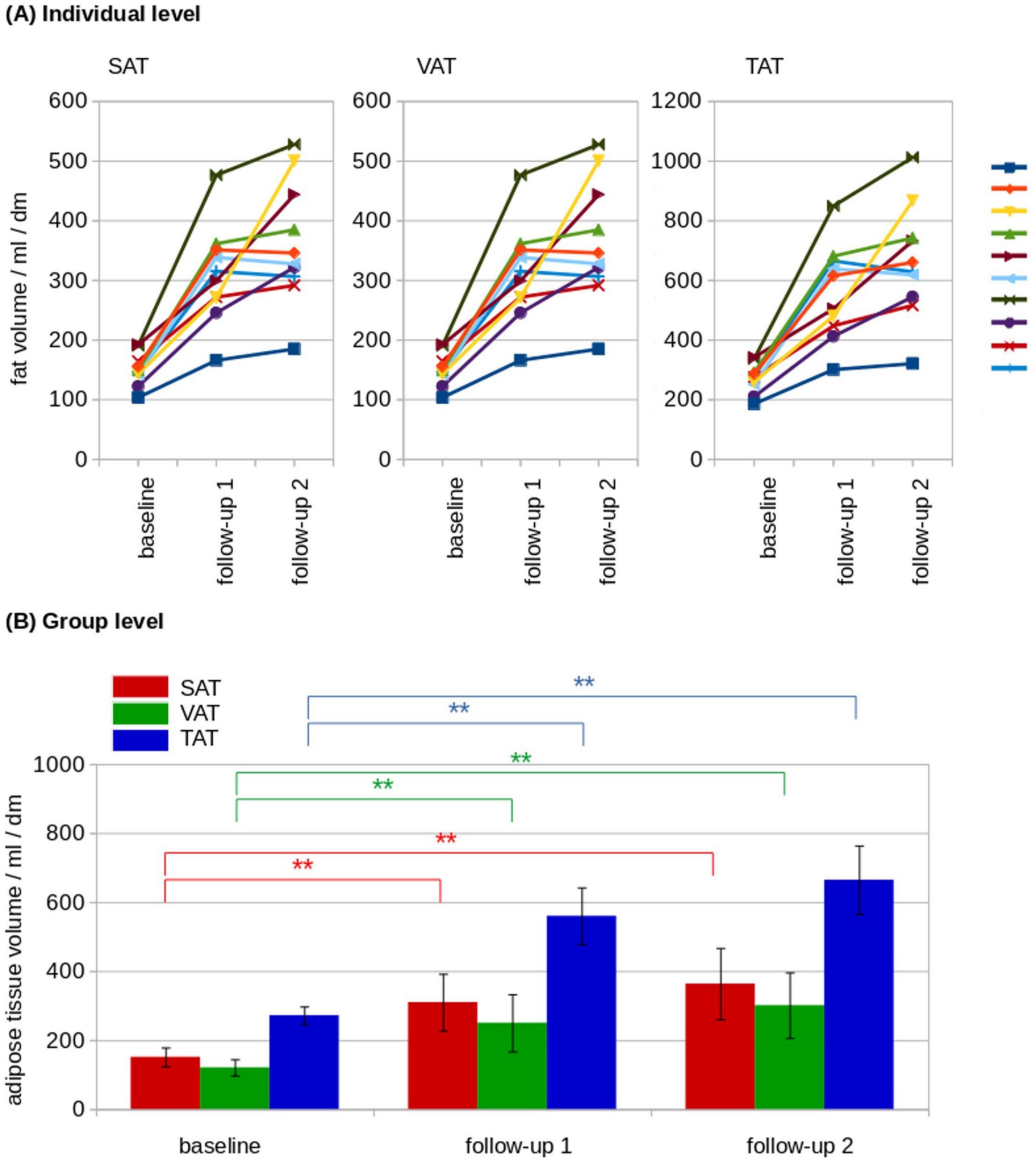
Longitudinal adipose tissue analysis. **(A)** At the individual level, normalized adipose tissue volumes showed a constant increase from baseline to follow-up 2 in subcutaneous adipose tissue (SAT), visceral adipose tissue (VAT), and total adipose tissue (TAT). The individual cats are indicated by the lines of different colors. **(B)** At the group level, adipose tissue volumes showed an increase of SAT (red), VAT (green) and TAT (blue). dm = 0.1 m; ^*^p < 0.01; ^**^p < 0.001.

**Figure 4:**
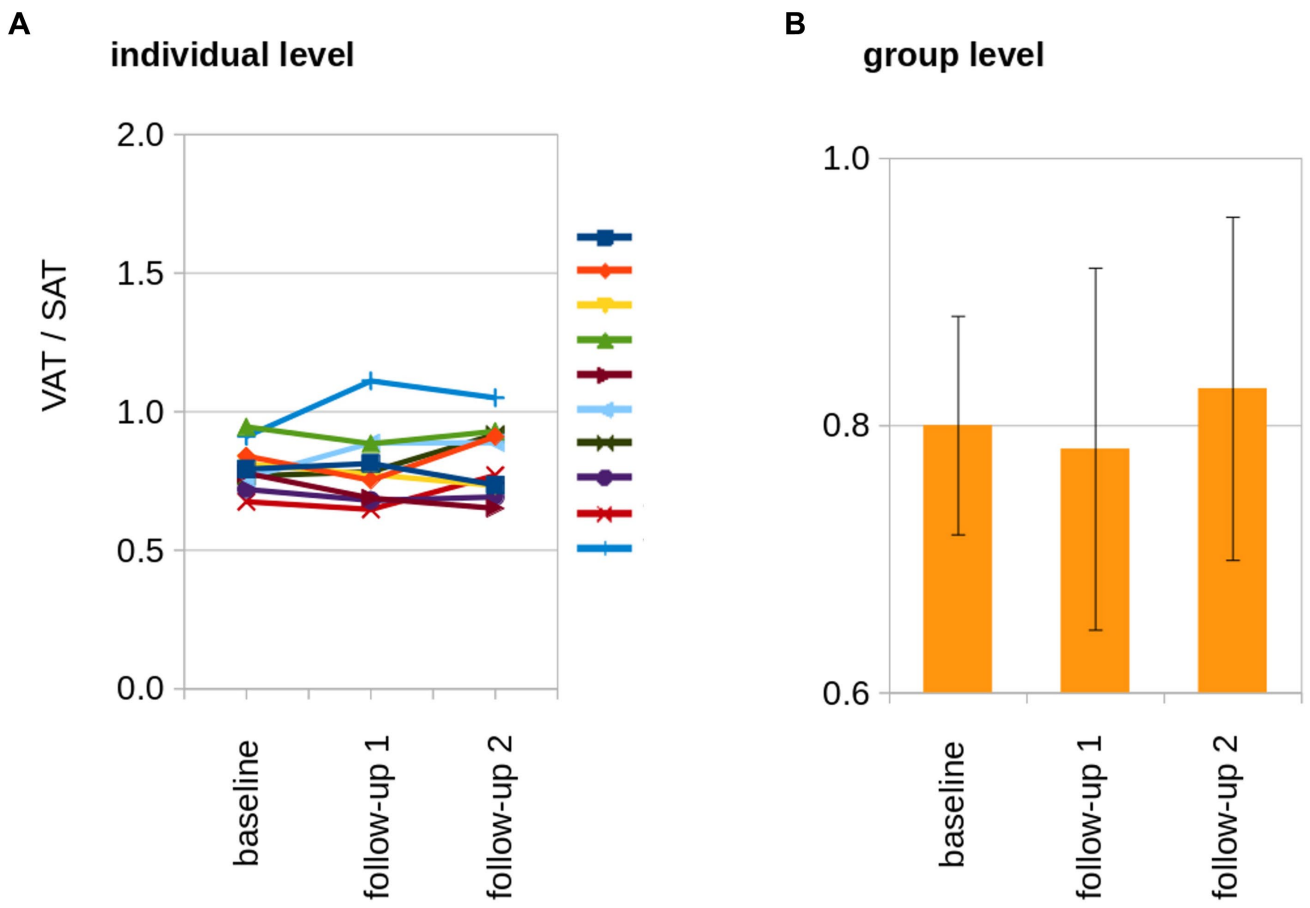
The longitudinal development of the VAT/SAT ratio. **(A)** At the individual level, the ratio of VAT/SAT was longitudinally almost constant in all cats. The individual cats are indicated by the lines of different colors. **(B)** At the group level, no increased VAT/SAT ratio from baseline to follow-up 2 could be observed.

SAT and VAT volumes were determined from MRI data and SAT and VAT were normalized by the length of the analyzed volumen (resulting in mL/dm). At the individual level, normalized adipose tissue volumes increased from baseline to follow-up 2 (Figure 3A). The normalized resulting volumes were then group averaged for baseline, follow-up 1, and follow-up 2. Here, adipose tissue volumes showed an increase for SAT, VAT, and total adipose tissue (TAT, TAT = SAT + VAT; Table 1; Figure 3B). The ratio of VAT/SAT was longitudinally almost constant in all cats from baseline to follow-up 2 (Figure 4A). Thus, also at the group level, no increased VAT/SAT ratio from baseline to follow-up 2 could be observed (Figure 4B).

**Table 1:** Adipose tissue volumes (SAT, VAT, and TAT in mL/dm) for baseline, follow-up 1 and follow-up 2 values are provided as median (interquartile range).

	Baseline (B)	Follow-up 1 (F1)	Follow-up 2 (F2)	*p* (B vs. F1)	*p* (F1 vs. F2)	*p* (B vs. F2)
SAT	130 (30)	243 (74)	288 (95)	0.0001^*^	0.3	0.0004^*^
VAT	151 (26)	291 (108)	375 (120)	0.0003^*^	0.3	0.0002^*^
TAT	280 (50)	563 (171)	660 (186)	0.0002^*^	0.3	0.0002^*^

Statistical significance indicated by *.

**Figure 5:**
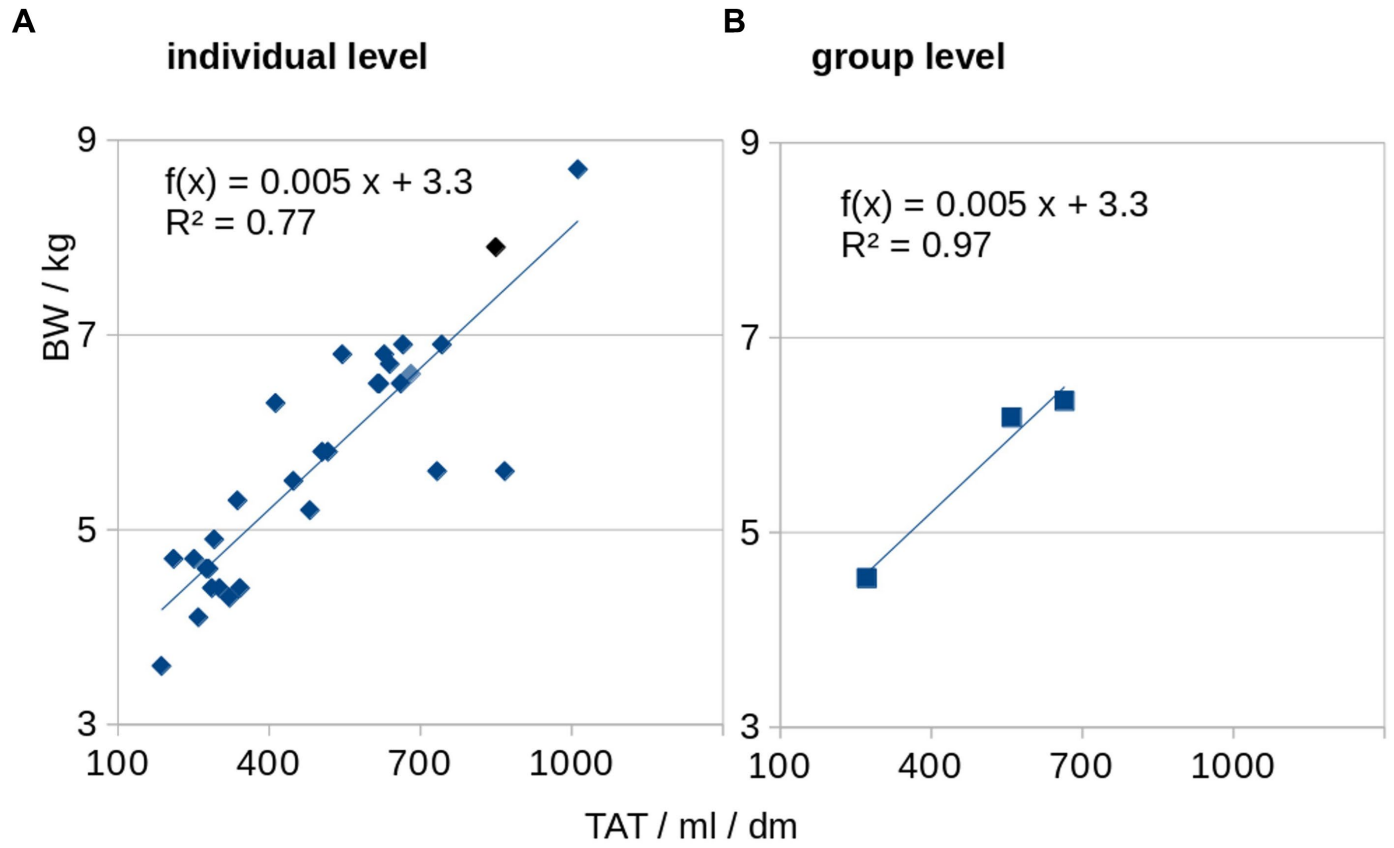
Association of total adipose tissue (TAT) and body weight (BW). BW and normalized TAT are associated at individual level **(A)** as well as at group level **(B)**.

**Figure 6:**
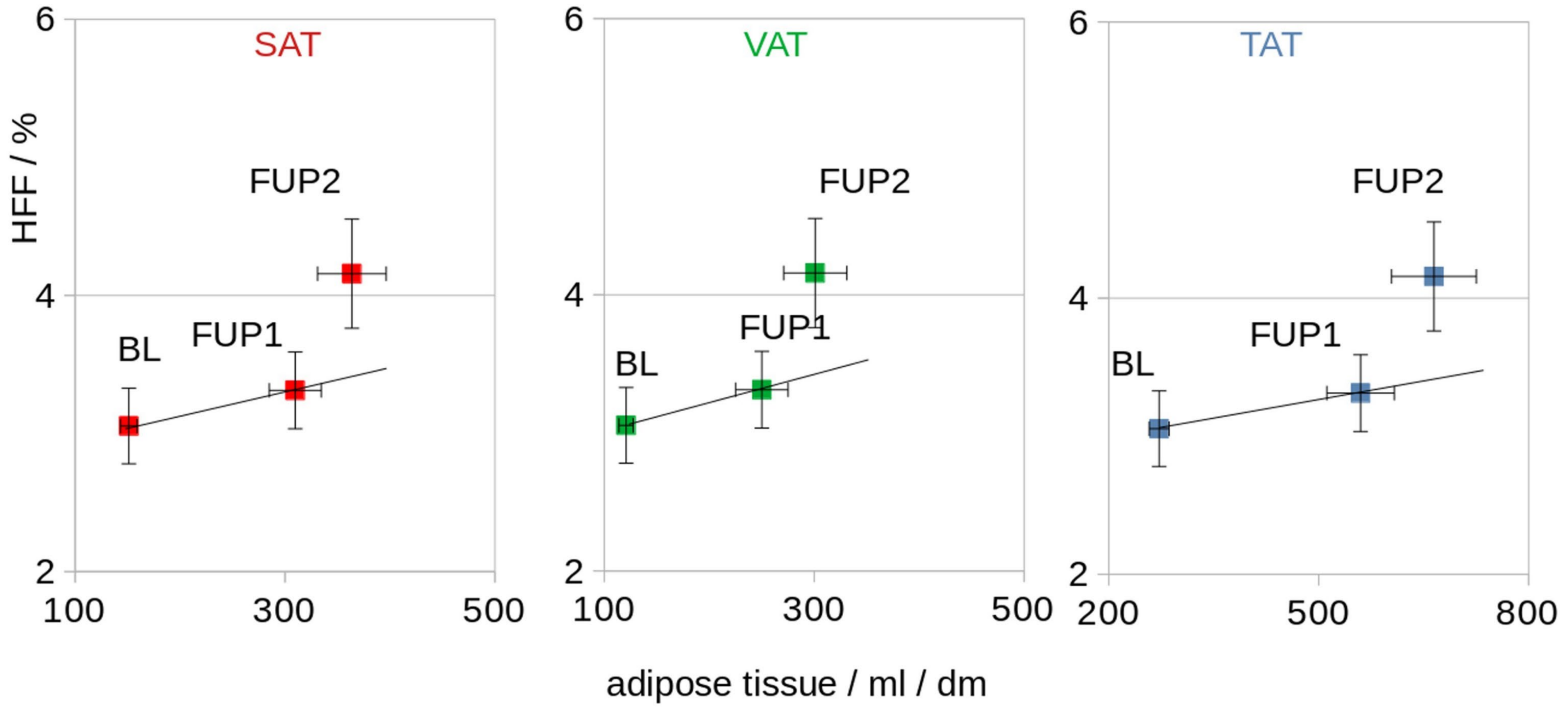
Association of the HFF and normalized adipose tissue volumes (SAT, VAT, and TAT) for baseline (BL), follow-up 1 (FUP1), and follow-up 2 (FUP2). The straight line indicates a proportional dependence (which is not observed for HFF). dm = 0.1 m.

BW and TAT showed a significant correlation and were associated at individual level (Spearman rank correlation R^2^ = 0.77, p = 0.005) as well as at group level (Pearson’s correlation R^2^ = 0.97, p < 0.001; Figure 5). The association of the HFF and normalized adipose soft tissue volumes (SAT, VAT, and TAT) for baseline, follow-up 1, and follow-up 2 showed that, with increasing adipose tissue volumes (i.e., VAT, SAT, and TAT, there were correlated with the BW), a disproportionare increase of HFF could be observed at follow-up 2 (Figure 4). From follow-up 2, despite a limited increase of BW and adipose tissue volumes (VAT, SAT, and TAT; Figure 4B), a more than proportional increase of HFF could be observed (Figure 6).

## 4. Discussion

In this study, the adipose tissue distribution in healthy adult cats was analyzed during a period of BW gain and a second period of consolidation of the gained BW as measured from whole-body MRI images. During this observation period, the different fat compartments (VAT, SAT, and TAT) were correlated with the HFF. The *ATLAS* algorithm was able to discriminate Dixon-MRI images from cats into fat compartments as a quantification of visceral and subcutaneous adipose tissue. MRI analysis of VAT, SAT, and TAT in 10 adult cats over 9 months revealed a significant increase in all fat compartments from baseline to follow-up 1, while there was no further significant increase from follow-up 1 to follow-up 2. Furthermore, the ratio VAT/SAT was almost constant from the baseline to follow-up 2, and always <1. The BW markedly increased during the first 20 weeks and much less during the following 20 weeks, approximately 39% and 2%, respectively. This was expected, because the dietary intervention aimed to stabilize the BW after the desired body condition score had been achieved. In fact, BW did not change significantly between follow-up 1 and 2. Similarly, significant SAT and VAT increase could be observed from MRI data, between baseline and follow-up 1, and between baseline and follow-up 2. The HFF, on the contrary, only mildly increased between the baseline and follow-up 1 and more consistently increased between follow-up 1 and 2 (almost 4 time more than during the first 20 weeks). From these results, it seems reasonable to assume that the adipose tissue tends to accumulate first subcutaneously and viscerally and only following a certain period of intake imbalance in the liver.

Companion animals, in particular cats and dogs, share the same living environment as humans, often similar lifestyle and are exposed to similar *noxae* (13). Moreover, their shorter life spam allows for the observation of the progression of diseases in shorter time compared to humans. Most diabetic cats have a type of diabetes mellitus that closely resembles human type 2 diabetes mellitus, and analogous to the situation in humans, genetical factors may predispose to diabetes mellitus as well as obesity and physical inactivity (20). For these reasons, an investigation of the pathophysiology of these diseases is important not only at clinical level for the single patients, but also in translational medicine.

In human medicine, there is considerable interest in identifying predictors for cardiometabolic risks and outcome in obesity-related pathologies. Amount and distribution of SAT and VAT have been considered promising predictors for that purpose (21). VAT has been linked to metabolic syndrome and cardiovascular mortality (7) and, conversely, VAT loss improves the inflammatory profile (22). A mean ratio of approximately 3.2/1 has been reported for SAT/VAT based on abdominal MRI images in chronically obese adult patients (23). Conversely, in our cat population, the amount of SAT and VAT was very similar at all the examined time points, with a median VAT/SAT ratio of about 0.8 at any analyzed time point. These data refer, however, to a relatively short period of observation. It remains unknown if the VAT/SAT ratio will change during chronicity of obesity on a longer term or during additional BW gain. However, by evaluating MRI images of only part of the abdomen, no difference in distribution of VAT and SAT has been found, neither in lean nor in obese cats (6). In 19 cats with mixed body condition, the VAT has been reported being higher than the SAT in 4 of the 5 analyzed body locations examined by computed tomography (15). In these studies, however, the image analysis does not include neither the entire abdomen nor the thorax, so that a complete quantification of VAT and SAT and their ratio is not possible. Moreover, regional differences in VAT and SAT distribution in different body regions (i.e., the thorax and the abdomen), as not been investigated.

In human literature, the biological effects of overfeeding have been widely investigated with hundreds of published articles (24). It has been reported that accumulation of intrahepatic triglycerides takes time, and follows fat deposition in the adipose tissue, accordingly to our results in cats. Research in humans, however, shows that the effect of overfeeding strongly depends on the composition of the diet and the magnitude of overfeeding (24). For example, carbohydrate overfeeding induces detectable liver fat increase in 3 weeks, detected with MR spectroscopy (2). In our research, micronutrients of the diet were not specifically modified, with the goal to mimic weight gain following merely increased food uptake. Different relationships between VAT/SAT and HFF are therefore possible with different dietary interventions.

Patterns of fat tissue distribution and their relationship are not very well understood even in human medicine, but special attention is paid to the HFF, because of its link with metabolic complication of obesity (25). Mesenteric fat thickness and visceral fat thickness assessed by ultrasound have been shown to be associated with fatty liver (26, 27), and the VAT/SAT ratio was elevated in parallel with the degree of fatty liver in young adults (28). In all these studies, thought, this association has not been quantified. In contrast, while the VAT and the SAT are strongly correlated to each other, both VAT and SAT are weakly correlated to HFF, based on MRI images analysis in normal-weight, overweight and obese children (29). Contrary to humans, where the VAT has been described to have major influence on risk factors for the metabolic syndrome (30), in cats, SAT seems to be more involved in the development of an inflammatory response (31). More recent research showed, however, that the HFF is even more strongly correlated with cardiometabolic disease in adults and young people than abdominal fat depots, as considered previously (32). Also in veterinary medicine, for this reason, investigation of the HFF in obesity-related disorders may be relevant for prognostic and therapeutic considerations. The estimation of HFF by ultrasonography is subjective, semiquantitative and has limited accuracy (28), and estimation of HFF by CT images may be inaccurate if the HFF is mild or moderate (33). These facts support the use of MRI for the investigation of obesity-related disorders, with the possibility to investigate, at the same time, abdominal fat and deposition of HFF. In the present study and in this analyzed cat model, results showed that a linear extrapolation of HFF from measurements of VAT and SAT may be inaccurate, leading to underestimation of HFF and that HFF, mainly in animals with long standing high body condition score, should be specifically investigated.

These findings have to be considered in the context that a gold standard is not available. Neither the HFF nor the VAT and SAT can be directly measured, absolute number are lacking, and the indirect measurements described are based on MRI. HFF based on MRI is an estimation of the true triglyceride hepatic content, which remains unknown. Due to the study design and the concurrent study, it was not possible to perform CT examination [as described previously (14, 15)] as validation or comparative method. Thus, no direct comparison with other techniques already established as a gold standard (i.e., dual energy X-ray absorptiometry) was possible. Another limitation is the small sample size and limited observation period, both of which were chosen out of consideration for animal welfare and a balance of interests. Compared to most of the studies of human medicine which investigate patients with non-alcoholic fatty liver disease, where the HFF can reach over 40%, the HFF of our population was relatively low (up to a median of 4.7%) and the subjects clinically healthy. Further studies on cats with higher body condition score and over a longer period, as well as investigation of feline patients with hepatic lipidosis, are needed to further investigate the relationships of SAT, VAT, TAT, and HFF and their clinical relevance. Lastly, increased lipid deposition in the muscle tissue, associated with feline obesity and insulin resistance (34), was not investigated in the present study. MRI field of view did not include the head and the extremities, considering the amount of fat tissue in these regions of minor relevance. Compared to humans, where the fat distribution is gender specific, no difference in fat distribution has been found in cats between gender in images of the abdomen only (6). Our study included only male and neutered cats, and we could investigate neither influence of gender and gonadal status, nor potential difference in fat distribution in body regions other than the abdomen and the thorax.

In conclusion, this study shows the feasibility of the already stablished algorithm, of quantifying fat tissue distribution also in cats. This method allows to document fat tissue deposition in cats based on MRI images, discriminating and quantifying VAT, SAT, and TAT. In adult cats during BW gain and stabilization of the gained BW, increased BW correlated to increased VAT, SAT and TAT, while VAT/SAT remained constant during the period of observation. At any time point, in the whole-body analysis, the SAT was more prominent that the VAT. This study of the correlation of VAT, SAT and HFF highlighted a more than proportional increase of the HFF during the 40 weeks of observation period. Despite equipment requirements, high costs, and need of general anesthesia in animals, MRI examination, with a combination of morphological images, functional sequences, and dedicated software solution, constitutes a very promising diagnostic, monitoring, and research tool in the investigation of obesity and correlated pathologies in cats.

## Data availability statement

The raw data supporting the conclusions of this article will be made available by the authors, without undue reservation.

## Ethics statement

The prospective, experimental study was approved by the Cantonal Veterinary Office, Ethics Committee, license number ZH118/16, was conducted in accordance with Animal Welfare Act of Switzerland.

## Author contributions

FC: conception and design, data acquisition, interpretation of data, drafting of the manuscript, and approved the submitted version. HR: design, data acquisition, interpretation of data, substantial revision of manuscript, and approved the submitted version. H-PM: data analysis, interpretation of data, drafting of manuscript, and approved the submitted version. JK: design, interpretation of data, drafting of manuscript, and approved the submitted version. All authors contributed to the article and approved the submitted version.

## Conflict of interest

The authors declare that the research was conducted in the absence of any commercial or financial relationships that could be construed as a potential conflict of interest.

## Publisher’s note

All claims expressed in this article are solely those of the authors and do not necessarily represent those of their affiliated organizations, or those of the publisher, the editors and the reviewers. Any product that may be evaluated in this article, or claim that may be made by its manufacturer, is not guaranteed or endorsed by the publisher.

## Abbreviations

BW: Body weight
GRE: Gradient recalled echo
HFF: Hepatic fat fraction
SAT: Subcutaneous adipose tissue
TAT: Total adipose tissue
VAT: Visceral adipose tissue
